# Subcapsular hepatic hematoma: a case of chronic expanding hematoma of the liver

**DOI:** 10.1186/s12876-021-01775-9

**Published:** 2021-05-27

**Authors:** Yasuyuki Ono, Shuji Kariya, Miyuki Nakatani, Yutaka Ueno, Takuji Maruyama, Atsushi Komemushi, Masaki Kaibori, Masatoshi Ikeda, Noboru Tanigawa

**Affiliations:** 1 Department of Radiology, Kansai Medical University, 2-5-1 Shinmachi, Hirakata, Osaka 573-1010 Japan; 2 Department of Surgery, Kansai Medical University, 2-5-1, Shinmachi, Hirakata, 573-1010 Osaka Japan; 3 Department of The Third Internal Medicine, Kansai Medical University, 2-5-1, Shinmachi, Hirakata, 573-1010 Osaka Japan

**Keywords:** Chronic expanding hematoma, Subcapsular hepatic hematoma, TAE

## Abstract

**Background:**

A chronic expanding hematoma (CEH) enlarges as a result of slight bleeding over several months, and the tissue shows a mixture of blood breakdown products, granulation tissue with capillary ingrowth, and inflammatory tissue. This report presents a case of a subcapsular hepatic CEH that was treated with transarterial embolization (TAE) and hepatectomy.

**Case presentation:**

A 56-year-old man presented with vomiting and right-sided abdominal pain. Plain abdominal computed tomography (CT) showed a high-density area of fluid collection beneath the capsule of the right hepatic lobe, which was diagnosed as a hematoma. From its anatomical position on the CT images, a subcapsular hepatic hematoma was diagnosed. Though conservative therapy was provided, CT-guided percutaneous drainage and TAE were performed due to worsening symptom. Because the patient's abdominal symptoms re-appeared, extended right segmentectomy including the hematoma was performed. In the resected specimen, the hematoma was located beneath the capsule of the right hepatic lobe, and it was displacing the hepatic parenchyma. Microscopic examination showed a thick fibrous capsule around the hematoma, peripheral lymphocyte and plasmacyte invasion, and aggregations of histiocytes containing phagocytosed hemosiderin.

**Conclusions:**

Anatomically, this was a case of a subcapsular hepatic hematoma, and pathologically it was shown to be a CEH. Complete surgical resection was effective treatment for this CEH.

## Background

Subcapsular hepatic hematomas caused by trauma or surgery differ from those caused by bleeding from the hepatic parenchyma because of the involvement of isolated arteries that have abundant communications with both intrahepatic and extrahepatic arteries [1]. Embolization of the intrahepatic and extrahepatic arteries is reportedly an effective treatment, and such hematomas are normally resorbed after hemostasis and disappear [1, 2].

A chronic expanding hematoma (CEH) enlarges as a result of slight bleeding over several months, and the tissue shows a mixture of blood breakdown products, granulation tissue with capillary ingrowth, and inflammatory tissue [3, 4].

Such CEHs may occur not only subdurally, but also in the limbs or mediastinum, but none have previously been reported in the liver. A CEH must be treated by complete resection of the hematoma, including the capsule [5–8].

A case of subcapsular hepatic CEH that was treated with transarterial embolization (TAE) and hepatectomy is reported.

## Case presentation

**Fig. 1:**
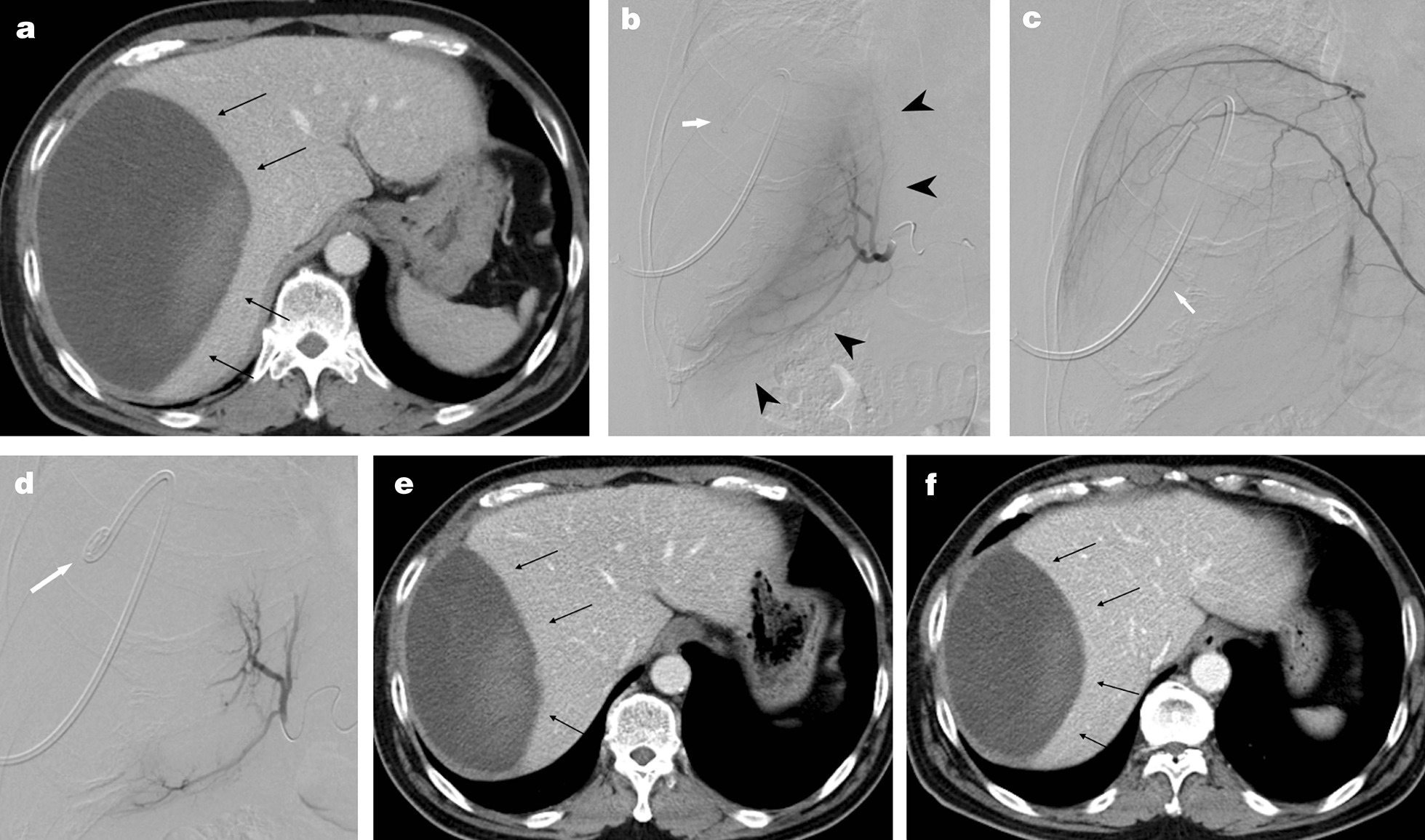
A 56-year-old man with a subcapsular hepatic hematoma diagnosed by abdominal computed tomography (CT). **a** Contrast-enhanced abdominal CT at the time of diagnosis. The hematoma is visible beneath the capsule of the right hepatic lobe (black arrows). **b**–**d** Percutaneous CT-guided drainage and transarterial embolization (TAE) are performed on Day 34 after the initial examination. **b** Digital subtraction angiography of the right hepatic artery. This artery is deviated, and the hepatic parenchyma is displaced by the hematoma (black arrowheads). The white arrow indicates the drainage catheter. There is no sign of contrast medium extravasation or an isolated artery. **c** Digital subtraction angiography of the right inferior phrenic artery. There is no sign of contrast medium extravasation or an isolated artery. **d** Digital subtraction angiography of the right hepatic artery after embolization. Blood flow is stopped in the periphery of the right hepatic artery. **e** Contrast-enhanced abdominal CT after percutaneous CT-guided drainage and TAE. The hematoma (black arrows) can be seen to have decreased in size. **f** Contrast-enhanced abdominal CT 1 month after TAE. The hematoma (black arrows) has not been resorbed, but it has started to regrow

A 56-year-old man presented with vomiting and right abdominal pain. Plain abdominal computed tomography (CT) showed a high-density area of fluid collection measuring 17.5 × 9.5 cm beneath the capsule of the right hepatic lobe, which was diagnosed as a hematoma (Fig. 1a). There were signs of compression of the hepatic parenchyma by the hematoma. Contrast-enhanced dynamic phase CT did not show any contrast enhancement within the hematoma or extravasation of contrast medium. There was no evidence of ascites. The vital signs at the outpatient visit were blood pressure 163/101 mmHg, heart rate 97 beats/min, SpO_2_ 100%, and respiratory rate 21 breaths/min. Blood test results were: white blood cell count 6400/µL, red blood cell count 332 × 10^4^/µL, platelet count 45.5 × 10^4^/µL, hemoglobin (Hb) 10.6 g/dL, aspartate aminotransferase 36 U/L, alanine aminotransferase 89 U/L, total bilirubin 0.4 mg/dL, albumin 4.0 g/dL, C-reactive protein 2.5 mg/dL, prothrombin time 87.2%, and activated partial thromboplastin time 30 s.

The patient had no history of trauma or of antiplatelet or anticoagulant drug use, and his medical history included nothing of note.

From its anatomical position on the CT images, a subcapsular hepatic hematoma was diagnosed. Conservative therapy was provided for 23 days, but the patient’s fever persisted, and his abdominal pain worsened. There was no change in the Hb level or progression of anemia during this time.

On Day 34 after the initial examination, CT-guided percutaneous drainage and TAE were performed to decompress the subcapsular hepatic hematoma and alleviate its symptoms. All procedures were performed using an interventional radiology-computed tomography/angiography system (AXIOM Artis dTA, Siemens Medical Solutions, Erlangen, Germany). To start with, a 10.2-Fr drainage catheter (Ultrathane, Dawson-Mueller Drainage Catheter, COOK Japan, Tokyo, Japan) was inserted, from which 500 mL of dark bloody fluid were drained. This reduced the size of the hematoma, but it did not disappear completely. After this decompression, TAE was performed. A 5-Fr, 25-cm-long sheath introducer (Super Sheath, Medikit, Tokyo, Japan) was inserted into the right femoral artery. A 5-Fr shepherd hook-shaped catheter (SHK1.0 Medikit) with a 2.2-Fr microcatheter (SIRABE, Piolax Medical Devices, Yokohama, Japan) was used.

Angiography of the right hepatic artery, the right inferior phrenic artery, and the right 8th-12th intercostal arteries, which were believed to be associated with the hematoma, was carried out (Fig. 1b, c). There was no extravasation of contrast medium from any of these vessels. The right hepatic artery, the right inferior phrenic artery, and each of the right intercostal arteries were embolized with 1-mm gelatin sponge particles (Serescue, Nippon Kayaku Co, Ltd., Tokyo, Japan). The total amount of gelatin sponge used was one sheet of 25 × 25 × 10 mm. The endpoint of embolization was the cessation of blood flow in the artery concerned (Fig. 1d). There were no complications as a result of either the percutaneous drainage or the TAE.

The drainage catheter was removed on Day 7 after embolization. The hematoma had shrunk to 13.7 × 7.5 cm, and the symptoms had improved, but it had not disappeared completely (Fig. 1e). The patient was discharged on Day 11 after TAE.

**Fig. 2:**
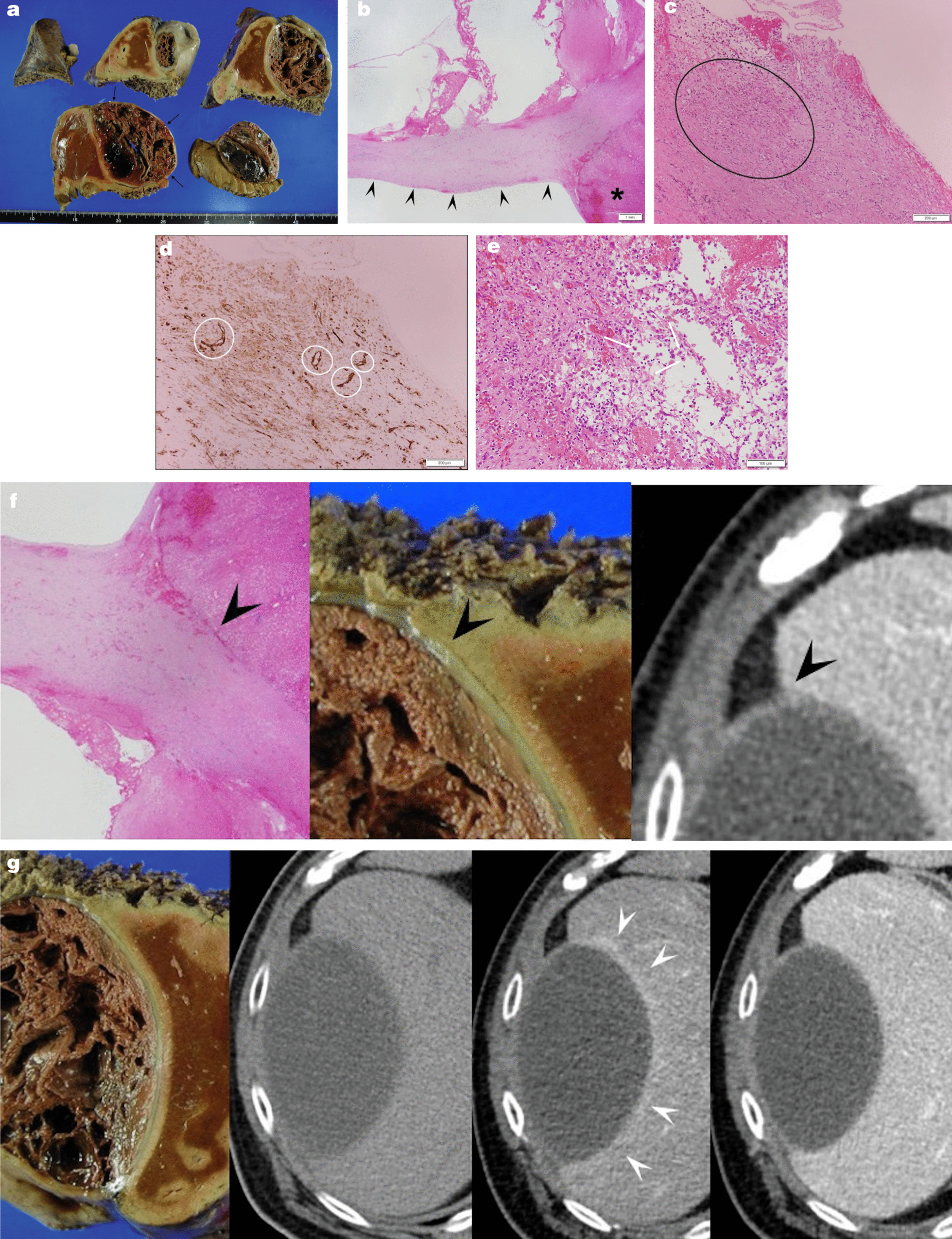
Extended right segmentectomy was performed on Day 54 after initial examination. **a** The resected specimen. A hematoma (black arrows) displacing the right lobe is visible beneath the capsule. **b** Magnified image of liver parenchyma and subcapsular hematoma. A hematoma with a fibrous capsule (black arrowheads) continuous with the liver parenchyma (asterisk) is seen. **c** Magnified image directly beneath the capsule. Inflammatory cell infiltration (black circle) is seen directly beneath the capsule. **d** Immunohistochemistry image of CD-31 antibody staining. Neocapillaries (white circle) with stained vascular endothelial cells are seen beneath the capsule. **e** Magnified image of the liver interior directly beneath the hematoma. Lymphocyte infiltration and siderophages (white arrows) are evident. **f** Contrast between a pathological specimen of the capsule and a computed tomography image. A thick fibrous capsule (black arrowhead) is seen on computed tomography, and in the contrast-enhanced delayed phase the contrast enhancement is weaker than in the liver parenchyma. **g** Contrast between the resected specimen and the preoperative images. The computed tomography images from the plain phase, arterial phase, and delayed phase are shown from the left. In the arterial phase, a perilesional parenchymal enhancement (white arrowhead) is seen

A CT scan 1 month after TAE showed that the hematoma now measured 13.3 × 8.2 cm, and it was growing again, rather than being resorbed (Fig. 1f). The patient’s abdominal symptoms also re-appeared. Extended right segmentectomy including the hematoma was performed as curative therapy on Day 54 after the initial examination. In the resected specimen, gross findings showed formation of a subcapsular hematoma of 11 × 9 cm in the right hepatic lobe and exclusion of the liver parenchyma (Fig. 2a). On microscopic examination, the hematoma was seen to have a thick fibrous capsule in contact with liver parenchyma (Fig. 2b). Lymphocyte and plasma cell invasion was seen around the capsule (Fig. 2c). On immunohistochemistry with CD-31 antibody staining, neocapillaries with stained vascular endothelial cells were seen beneath the capsule (Fig. 2d). In magnified images of the hematoma periphery, invasion of lymphocytes and aggregation of hemosiderin-laden histiocytes were seen (Fig. 2e). There was no evidence of vasculitis or vascular malformation, a tumorous lesion, or amyloidosis. The hematoma that developed beneath the capsule was a CEH, based on the clinical course, which was a chronic course, and the pathological findings.

One year after resection of the hematoma, there was no postoperative recurrence of the hematoma.

## Discussion and conclusions

Anatomically, this was a case of a subcapsular hepatic hematoma, and pathologically it was shown to be a CEH.

On the basis of previous reports, it was initially believed to be a subcapsular hepatic hematoma associated with an isolated artery, and TAE was therefore performed, but this proved ineffective [1, 9]. Its gradual increase in size during the clinical course meant that it was considered a CEH occurring beneath the hepatic capsule, and surgical resection was therefore carried out in line with past reports of CEHs at sites other than the liver [5–8]. Pathological signs of CEH were also evident, and this was therefore diagnosed as CEH beneath the hepatic capsule.

Most CEHs start out as very small traumatic hematomas that are not resorbed over time, but instead exhibit chronic growth. Pathologically, hematomas have a peripheral wall of dense fibrous tissue with a central space containing fresh and altered blood [10]. A chronic inflammatory response occurs within the hematoma, and neocapillaries are generated by the activation of potent local vasoactive substances. The activation of the fibrinolytic system in situ also causes cavitation of the hematoma. Blood leaks into this cavity from the neocapillaries, and the hematoma gradually grows in size. In the present patient, the cause of the initial hematoma formation was unknown, but its gradual expansion over time was consistent with previous reports, and signs diagnostic of CEH were also evident in the postoperative pathological specimen.

On CT imaging, cystic tumors and intracystic hemorrhage are considered in the differential diagnosis. There was no contrast enhancement in the interiors of the masses, and no solid components were seen; therefore, it was not diagnosed as a cystic tumor. The contrast between the resected specimen and the CT images was investigated. A thick capsule structure that was consistent with the fibrous capsule of a hematoma was seen on CT (Fig. 2f). The contrast enhancement of capsule was weaker than in the surrounding parenchyma. Moreover, in the contrast-enhanced arterial phase, a perilesional parenchymal enhancement was seen (Fig. 2g). Pathologically as well, the main properties of hematomas are inflammatory changes, so this contrast enhancement is thought to have reflected the increased blood flow accompanying the inflammatory response around the hematoma. These are findings not seen in cystic bleeding.

This patient was initially diagnosed with a subcapsular hepatic hematoma on the basis of diagnostic imaging findings. According to previous studies, a subcapsular hepatic hematoma may be iatrogenic or caused by blunt trauma, hemolysis, elevated liver enzymes, low platelet syndrome, amyloidosis, or vasculitis, and it reportedly occurs secondary to these conditions and grows rapidly [9, 11–14]. It has also been suggested that isolated arteries may be implicated in the pathology of hematomas that exhibit short-term rapid growth [1, 9, 14]. These isolated arteries communicate with the right inferior phrenic artery, the superior falciform ligament artery, and the cystic artery, all of which are extrahepatic arteries, as well as the intrahepatic arteries [15]. TAE is reportedly effective for the treatment of acute subcapsular hepatic hematoma due to arterial bleeding from these arteries [1, 9]. In the current patient, however, although this was a subcapsular hepatic hematoma anatomically, its clinical course and pathology showed that it was a CEH, and TAE was probably unsuccessful because the bleeding was coming from neocapillaries generated by a chronic inflammatory response. Complete surgical resection of the hematoma, including the capsule, is required, and incomplete resection is believed to entail a high risk of recurrence [16].

In conclusion, the case of a patient with a CEH of the liver was presented. Although it was a subcapsular hepatic hematoma based on its anatomic position on diagnostic imaging, its clinical course, with gradual growth rather than sudden occurrence, and its pathological findings indicated that it was a CEH. In addition, complete surgical resection was effective treatment for this CEH.

## Data Availability

The datasets used during the current study are available from the corresponding author on reasonable request.

## Abbreviations

CEH: Chronic expanding hematoma
TAE: Transarterial embolization
CT: Computed tomography
Hb: Hemoglobin
